# The Spatial Distribution of Relative Corneal Refractive Power Shift and Axial Growth in Myopic Children: Orthokeratology Versus Multifocal Contact Lens

**DOI:** 10.3389/fnins.2021.686932

**Published:** 2021-06-09

**Authors:** Fan Jiang, Xiaopeng Huang, Houxue Xia, Bingqi Wang, Fan Lu, Bin Zhang, Jun Jiang

**Affiliations:** ^1^School of Ophthalmology and Optometry, Wenzhou Medical University, Wenzhou, China; ^2^College of Optometry, Nova Southeastern University, Fort Lauderdale, FL, United States; ^3^Eye Hospital, Wenzhou Medical University, Wenzhou, China

**Keywords:** orthokeratology, multifocal soft contact lens, corneal refractive power, axial length, myopia

## Abstract

**Purpose:**

To determine if the spatial distribution of the relative corneal refractive power shift (RCRPS) explains the retardation of axial length (AL) elongation after treatment by either orthokeratology (OK) or multifocal soft contact lenses (MFCLs).

**Methods:**

Children (8–14 years) were enrolled in the OK (*n* = 35) or MFCL (*n* = 36) groups. RCRPS maps were derived by computing the difference between baseline and 12-month corneal topography maps and then subtracting the apex values. Values at the same radius were averaged to obtain the RCRPS profile, from which four parameters were extracted: (1) Half_x and (2) Half_y, i.e., the x- and y-coordinates where each profile first reached the half peak; (3) Sum4 and (4) Sum7, i.e., the summation of powers within a corneal area of 4- and 7-mm diameters. Correlations between AL elongation and these parameters were analyzed by multiple linear regression.

**Results:**

AL elongation in the OK group was significantly smaller than that in the MFCL group (*p* = 0.040). Half_x and Half_y were also smaller in the OK group than the MFCL group (*p* < 0.001 each). Half_x was correlated with AL elongation in the OK group (*p* = 0.005), but not in the MFCL group (*p* = 0.600). In an analysis that combined eyes of both groups, Half_x was correlated with AL elongation (β = 0.161, *p <* 0.001).

**Conclusions:**

The OK-induced AL elongation and associated RCRPS Half_x were smaller than for the MFCL. Contact lenses that induce RCRPS closer to the corneal center may exert better myopia control.

## Introduction

The incidence of myopia has risen over the last several decades (Williams et al., 2015; Holden et al., 2016), particularly in East Asian countries where about 80% of the 18-year-olds are myopic (Rudnicka et al., 2016). Myopia is usually associated with excessive axial length (AL) elongation (Morgan et al., 2012), which increases the risk of ocular complications such as myopic maculopathy, retinal detachment, and glaucoma (Marcus et al., 2011; Flitcroft, 2012). Orthokeratology (OK) lenses and multifocal soft contact lenses (MFCLs) are the two most often used optical devices in the clinic to correct refractive error and to retard AL elongation (Huang et al., 2016; Kang, 2018). OK lenses have a reverse-geometry on the back surface that flattens the central zone of the cornea and steepens the mid-peripheral zone during overnight wear (Sridharan and Swarbrick, 2003). During the day, this altered corneal front surface induces myopic defocus on the peripheral retina (Charman et al., 2006; Queiros et al., 2010, 2018), and this may be a mechanism for myopia retardation (Smith et al., 2009; Benavente-Perez et al., 2014). In contrast, daytime MFCL wear directly imposes peripheral retinal myopic defocus when the lenses are worn (Sankaridurg et al., 2011; Allinjawi et al., 2016).

Recent studies suggested the importance of the spatial distribution of the peripheral myopic defocus in retarding axial growth. Liu and Wildsoet reported that two-zone bifocal spectacle lenses incorporating +5 diopter (D) of peripheral defocus, extending from 1.25 to 2.75 mm from the lens center to its periphery, were effective in retarding axial growth in chicks. However, more peripheral zones, beginning 3.25 mm from the lens center, had no effect (Liu and Wildsoet, 2011). A study in monkeys reported that lenses imposing myopic defocus close to the optic axis were more effective in inhibiting axial growth than those in which defocus was limited to the more peripheral regions, e.g., 20 degrees away from the optic axis (Smith et al., 2020).

In clinical studies of OK lenses, the change of corneal refractive power (CRP) could be captured by corneal topography. The difference in CRP before and after OK treatment was calculated as the CRP shift (CRPS). Subtracting the apex value from each point of the CRPS then derives the relative CRPS (RCRPS) that is related with the myopic shift in defocus on the peripheral retina (Kang and Swarbrick, 2013). Some studies have suggested that RCRPS is strongly associated with AL elongation (Zhong et al., 2014, 2015; Lee et al., 2018; Hu et al., 2019). However, existing studies used only simple measures, such as the maximum value of either the whole map (Lee et al., 2018) or along some axes (Zhong et al., 2014), or the summed value within a certain area (Zhong et al., 2015; Hu et al., 2019). Neither maximum nor summed value provides information on the spatial distribution of the RCRPS because lenses with different spatial distributions of RCRPS may have similar maximum and summed values. Therefore, in this study, we proposed a new index that we identify as “Half_x” to quantify the spatial distribution of the RCRPS. Furthermore, we then determine if variations in Half_x explain the variations in the retardation of AL elongation observed in children who underwent OK treatment.

Both OK lenses and MFCLs act by modifying the refractive power of the front optical surface. However, few studies have analyzed the axial retardation effect of MFCLs through the perspective of the RCRPS. Therefore, a second aim of the present study was to examine whether or not Half_x, the new index on RCRPS spatial distribution, could also be applied to MFCL wear. The analysis of such an index on OK and MFCL subjects as a combined group would provide a unified theoretic background to interpret the axial retardation effect. Moreover, the knowledge obtained from OK lens design could be utilized in MFCL design.

## Materials and Methods

### Subjects

Thirty-seven MFCL subjects were enrolled in this registered clinical trial^1^ (Registration number: ChiCTR-OOC-17012103). Thirty-seven OK subjects were from a previous study of OK lens safety. The inclusion criteria for both groups were as follows: 8 to 14 years of age, spherical equivalent (SE) from −1.00 D to −5.00 D, corneal astigmatism ≤1.50 D, best-corrected visual acuity greater than 20/25, no binocular vision dysfunction, no obvious angle kappa, no history of an OK lens or MFCL wear or any other myopia control treatments such as atropine, no application of atropine for cycloplegia during the past 30 days, no contact lens contraindications or related eye and systemic disease. All procedures complied with the Declaration of Helsinki, and the protocol was reviewed and approved by the Ethics Committee of the Eye Hospital of Wenzhou Medical University. All the examinations were conducted after the subjects, and their guardians fully understood and signed informed consent.

### Refraction

Cycloplegic autorefraction was performed at baseline. One drop of 0.5% proparacaine was instilled. One minute later, this was followed by two drops of 1% cyclopentolate, administered 5 min apart. Refractive results were converted to SEs, which were calculated as the spherical power plus 1/2 cylindrical power.

### Lens Fitting

In both groups, the lenses were fitted to both eyes according to the manufacturer’s guidelines. For the OK group, a four-zone reverse geometry lens (Euclid Systems Corp., Herndon, VA, United States) is composed of oprifocon A [DK: 90 × 10^–11^ (cm^2^/s) (mL O_2_/mL^∗^mmHg)] was used. The diameter of the lenses ranged from 10.2 to 10.6 mm. The lens consisted of a central base curve with a 6.2-mm optic zone, a 0.5-mm wide reverse curve, a 1.0 to 1.2-mm wide alignment curve, and a 0.5-mm wide peripheral curve. The subjects were instructed to wear them for at least 8 h every night. OK lens prescriptions were changed if the uncorrected visual acuity was less than 20/25 after regular wear for one month or unacceptable lens decentration was observed. For the MFCL group, a daily disposable soft contact lens (BioThin, Bio Optic Inc., Taiwan, China) made of ocufilcon D [DK: 19 × 10^–11^ (cm^2^/s) (mL O_2_/mL^∗^mmHg)] with a diameter of 14.2 mm and a base curve of 8.6 mm was used. The lenses were designed to have the spherical distance power at the central treatment zone of 0–3 mm diameter and a myopic defocus zone of 3–6 mm diameter (Huang et al., 2020). Subjects were asked to wear the MFCL for at least 5 days/week, 8 h/day. MFCL prescriptions were changed if the corrected visual acuity was less than 20/25, or the spherical over-refraction achieved −0.50 D in the follow-up visits.

### Axial Length

AL was measured by the IOL-Master system (IOL-Master, Carl Zeiss, Jena, Germany) at baseline and at the 12-month follow-up visit. Measurements were performed by the same operator, and five reliable readings with a signal-to-noise ratio >2.1 were collected, of which the median value was used for analysis.

### Corneal Topography

**FIGURE 1:**
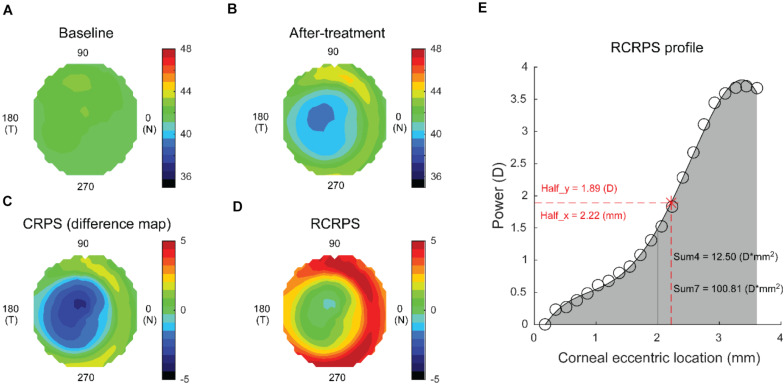
Computation of the RCRPS for a representative eye. **(A)** An axial map at baseline. **(B)** An axial map at 12 months after treatment. **(C)** CRPS was derived by subtracting the baseline map from the after-treatment map. **(D)** RCRPS was derived by subtracting the center point value from each point on the CRPS. **(E)** RCRPS profile derived by taking the mean of each ring. Data are for an eye wearing the OK lens. Similar determinations were made for eyes wearing the MFCLs. RCRPS, relative corneal refractive power shift; CRPS, corneal refractive power shift; OK, orthokeratology; MFCL, multifocal soft contact lens; Half_x, the corneal eccentric location where RCRPS reached the half peak value; Half_y, half of the RCRPS peak value; Sum4, the integrated value of the RCRPS located within an area of 4-mm diameter; Sum7, the integrated value of the RCRPS located within an area of 7-mm diameter.

Corneal topography (Medmont E300; Medmont International Pty. Ltd., Victoria, Australia) was obtained at baseline and 12 months after lens wear was initiated to quantify RCRPS. For the OK group, the 12-month measurement was performed with the lens off, and for the MFCL group, it was done with the lens on. All measurements were conducted between 8 and 10 am to minimize the diurnal variation. Each exported axial map had 32 rings, each containing 300 data points. After preparing the baseline and the after-treatment topography maps (Figures 1A,B respectively), the CRPS map (Figure 1C) was obtained by subtracting the baseline map data from the after-treatment map data. Then, the apex value was subtracted from each point to derive the RCRPS map (Figure 1D). The profile of the RCRPS was calculated by taking the mean value of each ring and fitting a polynomial curve through them (Figure 1E). The point where RCRPS reached the half-peak value was identified, and the x-axis value of this point was defined as the Half_x, indicating how fast the RCRPS had risen. The y-axis value of the point was defined as the Half_y. Sum4 was defined as the integrated value of the RCRPS located within a corneal area of 4-mm diameter. Similarly, Sum7 was defined as the integrated value of the RCRPS located within a corneal area of 7-mm diameter. All calculations were conducted using the custom MATLAB function (MathWorks, Natick, WA, United States).

Pupil size was extracted from the topographic data obtained under ambient mesopic room illumination, but photopic conditions were still considered due to topographer intrinsic light level (Periman et al., 2003). Pupil radius was calculated by halving the average of horizontal and vertical pupil diameters.

### Statistical Analyses

Only the right eyes were included in the analysis. The Schapiro-Wilk test was used to examine data distribution normality. For comparisons between the OK and MFCL groups, *t*-tests were used for normally distributed data, and Mann-Whitney *U* tests were used otherwise. Simple univariate regression was used first to explore the associations between AL elongation and baseline and RCRPS parameters. The parameters that showed significance were then further analyzed in a stepwise multivariate regression. Variable selection in the multivariable model was based on the Akaike Information Criterion (Akaike, 1974). All analyses were performed using the R programming package^2^ (version 3.6.3), and *p* < 0.05 was defined as statistically significant.

## Results

Two eyes of the OK group and one eye of the MFCL group were excluded due to poor measurement quality of the corneal topography. A total of 71 eyes were included in the analyses. There were no significant differences between the two groups in the baseline information (Table 1).

**TABLE 1:** Baseline information for subjects.

**Parameter**	**OK (*n* = 35)**	**MFCL (*n* = 36)**	***P* value**
Age (y)	10.5 ± 1.6	10.6 ± 1.4	0.953
Males (%)	12 (34%)	14 (39%)	0.876
SE (D)	−2.73 ± 0.99	−2.55 ± 0.86	0.515
Pupil radius (mm)	1.81 ± 0.48	1.83 ± 0.30	0.461

*OK, orthokeratology; MFCL, multifocal contact lens; y, years old; SE, spherical equivalent; D, diopter.*

### AL Elongation

The 12-month AL elongation for subjects in the OK group was 0.19 ± 0.20 mm, which was significantly smaller than in the MFCL group, 0.27 ± 0.15 mm (*p* = 0.040). The rate of the AL change was margin for the two groups (OK: 0.77%, MF: 1.12%, *p* = 0.049 (Table 2).

**TABLE 2:** Axial length and axial length change for subjects.

	**OK**	**MFCL**	***P* value**
Baseline (mm)	24.63 ± 0.65	24.50 ± 0.63	0.366
12 months (mm)	24.82 ± 0.55	24.77 ± 0.64	0.723
Change (mm)	0.19 ± 0.20	0.27 ± 0.15	0.040
Rate of change (%)	0.77 ± 0.86	1.12 ± 0.60	0.049

### RCRPS Profiles

**FIGURE 2:**
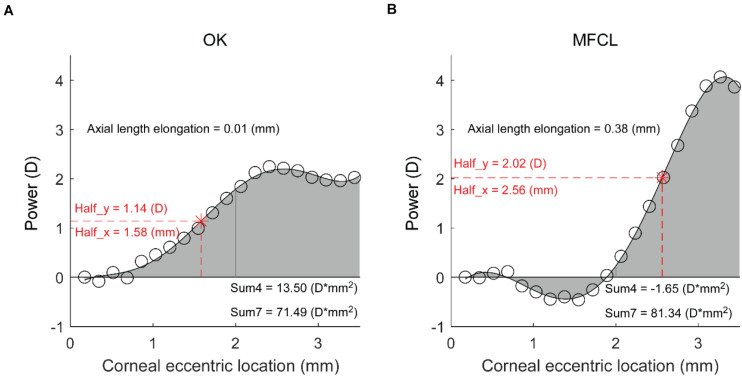
Representative RCRPS profiles. **(A)** An eye in the OK group. **(B)** An eye from the MFCL group. RCRPS, relative corneal refractive power shift; D, diopter; OK, orthokeratology; MFCL, multifocal soft contact lens; Half_x, the corneal eccentric location where RCRPS reached the half peak value; Half_y, half of the RCRPS peak value; Sum4, the integrated value of the RCRPS located within an area of 4-mm diameter; Sum7, the integrated value of the RCRPS located within an area of 7-mm diameter.

The RCRPS profiles for individuals in the OK and MFCL groups were both clearly S-shaped, as shown in representative profiles (Figures 2A,B respectively). However, there were significant differences between the two groups, as seen in the representative profiles. For instance, the profile for the OK subject (Figure 2A) reached the half peak at a relatively small corneal eccentric location (Half_x = 1.58 mm). At the 2.5 mm location, the value reached a plateau. In contrast, the profile for the MFCL subject (Figure 2B) remained low in the central region before it increased and reached the half peak farther from the center (Half_x = 2.56 mm) than for the OK subject. Consequently, the sum of values within the 4-mm diameter (Sum4 = 13.50 D^∗^mm^2^) for the OK subject was greater than for the MFCL subject (Sum4 = −1.65 D^∗^mm^2^), even though the two profiles had similar Sum7 values. Meanwhile, the AL elongation for the OK subject was much smaller than for the MFCL subject.

**FIGURE 3:**
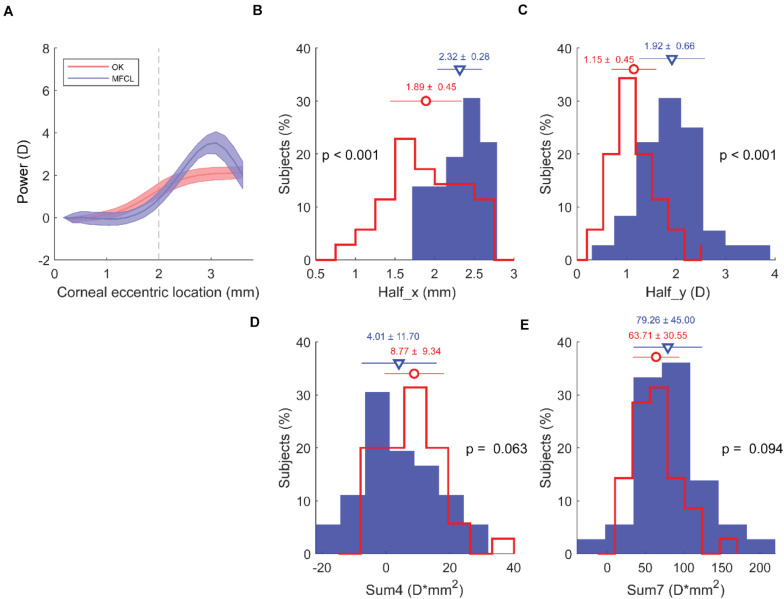
RCRPS data from all subjects. **(A)** RCRPS profiles for the OK group (red) and MFCL group (blue). Solid line: mean value; shaded area: 95% confidence interval for the population means. **(B)** Half_x, **(C)** Half_y, **(D)** Sum4, and **(E)** Sum7. **(B–E)** OK group, red line; MFCL group, blue bars. RCRPS, relative corneal refractive power shift; D, diopter; OK, orthokeratology; MFCL, multifocal soft contact lens. Half_x, the corneal eccentric location where RCRPS reached the half peak value; Half_y, half of the RCRPS peak value; Sum4, the integrated value of the RCRPS located within an area of 4-mm diameter; Sum7, the integrated value of the RCRPS located within an area of 7-mm diameter.

The RCRPS profile for the entire OK group started to rise at a location closer to the center than for the entire MFCL group (Figure 3A). The Half_x for the OK group (1.89 ± 0.45 mm) was significantly smaller than for the MFCL group (2.32 ± 0.28 mm; *p* < 0.001; Figure 3B). The Half_y for the OK group (1.15 ± 0.45 D) was significantly smaller than for the MFCL group (1.92 ± 0.66 D; *p* < 0.001; Figure 3C). The Sum4 in the OK group (8.77 ± 9.34 D^∗^mm^2^) was greater than for the MFCL group (4.01 ± 11.70 D^∗^mm^2^), but the difference was not significant (*p* = 0.063; Figure 3D). The Sum7 was also not significantly different between the two groups (OK: 63.71 ± 30.55 D^∗^mm^2^ vs MFCL: 79.26 ± 45.00 D^∗^mm^2^, *p* = 0.094; Figure 3E).

In the OK group, all of the RCRPS parameters, including Half_x (*r* = 0.428, *p* = 0.010), Half_y (*r* = −0.412, *p* = 0.014), Sum4 (*r* = −0.534, *p* < 0.001), and Sum7 (*r* = −0.477, *p* = 0.004) were correlated with the baseline SE. None of these parameters were correlated with the baseline SE in MFCL subjects.

### Association Between AL Elongation and RCRPS Parameters

**FIGURE 4:**
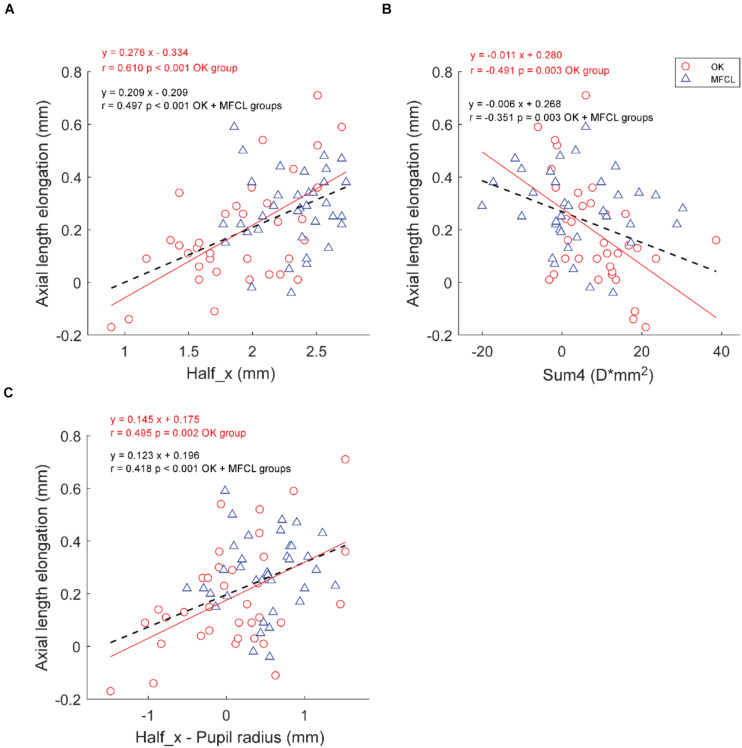
Association between axial length elongation and RCRPS parameters **(A)** Half_x, **(B)** Sum4, **(C)** Half_x – Pupil radius for the OK group (red circles) and the MFCL group (blue triangles). The red solid line is the regression line of the OK data; the black dashed line is the regression line of the combined data. RCRPS, relative corneal refractive power shift; D, diopter; OK, orthokeratology; MFCL, multifocal soft contact lens; combined data: OK and MFCL; Half_x, the corneal eccentric location where RCRPS reached the half peak value; Sum4, the integrated value of the RCRPS located within an area of 4-mm diameter.

The Half_x was positively correlated with AL elongation in the OK group and in the group formed by the combination of OK and MFCL subjects, but not the MFCL group (OK: *r* = 0.610, *p* < 0.001; MFCL: *r* = 0.090, *p* = 0.600; Combined data: *r* = 0.497, *p* < 0.001; Figure 4A). The Sum4 was negatively correlated with AL elongation in the OK group (*r* = −0.491, *p* = 0.003) and the combined group (*r* = −0.351, *p* = 0.003), but not the MFCL group MFCL group (*r* = −0.146, *p* = 0.395, Figure 4B). The difference between Half_x and pupil radius was positively correlated with AL elongation in the OK group (*r* = 0.495, *p* = 0.002) and the combined group (*r* = 0.418, *p* < 0.001), but not the MFCL group (*r* = 0.102, *p* = 0.554, Figure 4C). For both the OK and MFCL groups, the Half_y and Sum7 were not correlated with AL elongation (Table 3).

**TABLE 3:** Linear regression analysis of AL elongation association with baseline data and RCRPS parameters.

**Group**	**Parameter**	**Univariate model**		**Multivariate model**	
		**Beta (95% CI)**	***P* value**	**Beta (95% CI)**	***P* value**
OK	Age (year)	−0.070 (−0.106 to −0.034)	**0.001**	−0.044 (−0.078 to −0.011)	**0.014**
	Gender (boys)	0.035 (−0.105 to 0.174)	0.628	\	\
	Baseline AL (mm)	−0.189 (−0.275 to −0.103)	**<0.001**	−0.066 (−0.161 to 0.029)	0.183
	Baseline SE (D)	0.072 (0.006 to 0.138)	**0.040**	Removed	
	Half_x (mm)	0.276 (0.154 to 0.398)	**<0.001**	0.186 (0.064 to 0.309)	**0.005**
	Half_y (D)	−0.037 (−0.189 to 0.116)	0.640	\	\
	Sum4 (D*mm^2^)	−0.011 (−0.017 to −0.004)	**0.003**	Removed	
	Sum7 (D*mm^2^)	−0.002 (−0.004 to 0.000)	0.114	\	\
	Half_x – PR (mm)	0.145 (0.058 to 0.231)	**0.002**	Removed	
MFCL	Age (year)	−0.045 (−0.076 to −0.014)	**0.008**	−0.045 (−0.076 to −0.014)	**0.008**
	Gender (boys)	0.016 (−0.086 to 0.118)	0.757	\	\
	Baseline AL (mm)	−0.015 (−0.092 to 0.062)	0.710	\	\
	Baseline SE (D)	0.013 (−0.044 to 0.069)	0.666	\	\
	Half_x (mm)	0.046 (−0.126 to 0.219)	0.600	\	\
	Half_y (D)	−0.014 (−0.087 to 0.060)	0.716	\	\
	Sum4 (D*mm^2^)	−0.002 (−0.006 to 0.002)	0.395	\	\
	Sum7 (D*mm^2^)	0.000 (−0.001 to 0.001)	0.453	\	\
	Half_x – PR (mm)	0.033 (−0.075 to 0.141)	0.554	\	\
Combined data	Age (year)	−0.058 (−0.083 to −0.034)	**<0.001**	−0.045 (−0.067 to −0.023)	**<0.001**
	Gender (boys)	0.019 (−0.069 to 0.107)	0.668	\	\
	Baseline AL (mm)	−0.109 (−0.171 to −0.048)	**0.001**	−0.047 (−0.102 to 0.007)	0.095
	Baseline SE (D)	0.050 (0.006 to 0.095)	**0.031**	Removed	
	Half_x (mm)	0.209 (0.123 to 0.295)	**<0.001**	0.161 (0.082 to 0.241)	**<0.001**
	Half_y (D)	0.022 (−0.040 to 0.084)	0.485	\	\
	Sum4 (D*mm^2^)	−0.006 (−0.010 to −0.002)	**0.003**	Removed	
	Sum7 (D*mm^2^)	−0.001 (−0.002 to 0.000)	0.290	\	\
	Half_x – PR (mm)	0.123 (0.060 to 0.186)	**<0.001**	Removed	

*CI, confidence interval; AL, axial length; RCRPS, relative corneal refractive power shift; SE, spherical equivalent, D, diopter; OK, orthokeratology; MFCL, multifocal soft contact lens; Combined data, OK and MFCL groups together; Half_x, the corneal eccentric location where RCRPS reached the half peak value; Half_y, half of the RCRPS peak value; Sum4, the integrated value of the RCRPS located within an area of 4-mm diameter; Sum7, the integrated value of the RCRPS located within an area of 7-mm diameter; PR, pupil radius; Removed, Based on the Akaike Information Criterion, the factor was not selected for analysis in the multivariate model. Bolded values indicate *p* < 0.05.*

Regarding baseline parameters, age, AL, and SE were correlated with AL elongation in the OK group and the combined group, while only age was correlated with AL elongation in the MFCL group (Table 3). Stepwise multiple regression analyses showed that in the OK group and the combined group, only age and Half_x were significantly correlated with AL elongation. Decreasing Half_x by 1 mm was associated with a 0.161 mm reduction in 12-month AL elongation after optical interventions (*p* < 0.001).

## Discussion

In the present study, we found that AL elongation and the new index Half_x in the OK group were significantly smaller than that in the MFCL group. More importantly, multiple regression analyses revealed that Half_x was significantly correlated with AL elongation in the OK group and in the combined OK and MFCL groups, but not the MFCL group alone.

The key difference in RCRPS profile could be explained by the differences in how MFCL and OK lenses alter the optical surfaces. For the MFCL, the changes in optical surfaces are mainly set by the lens design, which manifests as a sharply rising edge in the MFCL profile. For the OK lens, the changes in optical surfaces depend not only on lens design but also on the response of the cornea. With the optical zone pressing the central portion of the cornea and the alignment zone fitting closely to the peripheral corneal surface, a negative pressure forms in the reverse zone (VanderVeen et al., 2019). In the central portion of the cornea, the epithelial layer becomes significantly thinned while the stromal layer shows little change. Moving away from the center, the epithelial thinning gradually disappears while the stromal layer significantly thickens (Alharbi and Swarbrick, 2003; Lian et al., 2013). Such a gradual change from the center to mid-periphery was consistent with the RCRPS profile for the OK group in the current study, a gradient rising gradually from the center to the reverse zone. Because the RCRPS profile in OK subjects depends on the reaction of the cornea, all of the RCRPS parameters were correlated with baseline SE in the OK group but not in the MFCL group.

AL elongations of the OK and MFCL groups, 0.19 mm and 0.27 mm respectively, were consistent with the previous study (Paune et al., 2015). Our results suggest that the spatial distribution of the RCRPS, rather than the total amount (Zhong et al., 2015; Hu et al., 2019), is critical in slowing myopia progression. First, AL elongation was not related to the peak values, and the significantly greater peak values in MFCL subjects did not lead to smaller AL elongations. Second, the observation that Sum4 was correlated with AL elongation, while Sum7 was not, indicates that myopic defocus in the far periphery may not be as crucial as the myopic defocus in the paracentral periphery. Third, in the multiple regression analyses, the Half_x remained significant while Sum4 did not. The Half_x was correlated with AL elongation for the OK group but not for the MFCL group. One interpretation was that the RCRPS in the MFCL group was too far from corneal center to get into pupil region. Lights that originally could induce myopic defocus in the peripheral retina were partially blocked by iris. The other interpretation was the wearing time. OK lenses were used every night and the defocus treatments were in place all day long. MFCLs were requested to wear 5 days/week, 8 h/day, and the defocus treatments disappeared with the lenses off. Lam et al. reported that wearing time was a contribution factor to retardation effect of myopia progression with defocus incorporated soft contact lenses (Lam et al., 2014).

Previous studies have directly analyzed the summation of RCRPS components. Our results agreed with Hu et al., who reported that the area sum of RCRPS within a 4-mm diameter was correlated with AL elongation during OK lens wear (Hu et al., 2019). However, our study did not support previously identified predictive factors such as the maximal value and the sum of RCRPS within a 7.2-mm diameter (Zhong et al., 2015). Although there are no previous studies directly examining the spatial distribution of RCRPS, some studies did indicate an association between AL elongation and spatial distribution in an indirect way. A recent study showed that a smaller back optic zone diameter for the OK lens would produce a smaller plus power ring diameter on the cornea’s front surface. When the plus power ring’s horizontal sector was inside the pupil, the mean AL elongation of those subjects was 76% lesser than the subjects who had the plus power ring outside the pupil (Paune et al., 2021). Our study supported this result and found a linear correlation between AL elongation and the amount of Half_x inside the pupil. One study compared the reduction of the peripheral retinal hyperopic defocus in two different designs of progressive MFCLs. Only the lens with the additional power starting at the 3.5-mm diameter caused a significant reduction in the peripheral retinal hyperopic defocus, while the lens with the additional power starting at the 5.0-mm diameter did not (Allinjawi et al., 2016). Li et al. also reported that MFCLs with two concentric defocus rings in the periphery had better myopia control than MFCLs with a single peripheral defocus ring (Li et al., 2017). Due to the presence of the two concentric defocus rings in the periphery, the rising edge of the inner ring may be closer to the center than that in MFCLs with only one defocus ring. Smith et al. (2020) compared the treatment effects in infant monkeys with those in human clinical trials. They suggested that the relative effects on myopia control of the different optical treatments in both monkeys and humans were dependent on the eccentricity of the defocus.

The association between AL elongation and Half_x looks promising but should be taken with caution. First, in the current study, we only analyzed one design of MFCL. The effect of various optical designs of MFCLs on the spatial distribution of RCRPS should be compared in future studies. Second, other potentially influencing factors such as lens wearing time, and near-working time should be incorporated into the multiple regression analyses.

## Conclusion

AL elongation in the OK group was significantly smaller than in the MFCL group over the 12-month study period. The difference in AL elongation may be attributed to the different RCRPS profiles induced by the two treatment modalities. Contact lenses with a smaller center treatment zone and closer adjacent additional power may lead to better myopia control.

## Data Availability Statement

The raw data supporting the conclusions of this article will be made available by the authors, without undue reservation.

## Ethics Statement

The studies involving human participants were reviewed and approved by the Ethics Committee of the Eye Hospital of Wenzhou Medical University. Written informed consent to participate in this study was provided by the participants’ legal guardian/next of kin.

## Author Contributions

FL, BZ, and JJ conceived the experiments and modified the manuscript. FJ and BZ determined the experimental methods, analyzed the data, and interpreted the data. XH, HX, and BW performed the experiments. FJ wrote the manuscript. All authors contributed to manuscript revision, read, and approved the submitted version.

## Conflict of Interest

The authors declare that the research was conducted in the absence of any commercial or financial relationships that could be construed as a potential conflict of interest. The reviewer ZC declared a past co-authorship with one of the author, JJ, to the handling editor.

## Footnotes

1 http://www.chictr.org.cn/index.aspx

2 HTTP://www.r-project.org/
